# Metalloproteinases in Restorative Dentistry: An In Silico Study toward an Ideal Animal Model

**DOI:** 10.3390/biomedicines11113042

**Published:** 2023-11-14

**Authors:** Simone Gomes de Oliveira, Nelson Kotowski, Helio Rodrigues Sampaio-Filho, Flávio Henrique Baggio Aguiar, Alberto Martín Rivera Dávila, Rodrigo Jardim

**Affiliations:** 1 Piracicaba School of Dentistry, Campinas State University, Piracicaba 13414-903, SP, Brazil; 2 School of Dentistry, State University of Rio de Janeiro, Rio de Janeiro 20551-030, RJ, Brazil; 3 Computational and Systems Biology Laboratory, Oswaldo Cruz Institute, Oswaldo Cruz Foundation, Rio de Janeiro 21040-900, RJ, Brazil; nelson.filho@ioc.fiocruz.br (N.K.); alberto.davila@fiocruz.br (A.M.R.D.);

**Keywords:** animal model, phylogeny, restorative dentistry

## Abstract

In dentistry, various animal models are used to evaluate adhesive systems, dental caries and periodontal diseases. Metalloproteinases (MMPs) are enzymes that degrade collagen in the dentin matrix and are categorized in over 20 different classes. Collagenases and gelatinases are intrinsic constituents of the human dentin organic matrix fibrillar network and are the most abundant MMPs in this tissue. Understanding such enzymes’ action on dentin is important in the development of approaches that could reduce dentin degradation and provide restorative procedures with extended longevity. This in silico study is based on dentistry’s most used animal models and intends to search for the most suitable, evolutionarily close to *Homo sapiens*. We were able to retrieve 176,077 mammalian MMP sequences from the UniProt database. These sequences were manually curated through a three-step process. After such, the remaining 3178 sequences were aligned in a multifasta file and phylogenetically reconstructed using the maximum likelihood method. Our study inferred that the animal models most evolutionarily related to *Homo sapiens* were *Orcytolagus cuniculus* (MMP-1 and MMP-8), *Canis lupus* (MMP-13), *Rattus norvegicus* (MMP-2) and *Orcytolagus cuniculus* (MMP-9). Further research will be needed for the biological validation of our findings.

## 1. Introduction

Animal models are used to investigate the efficacy and safety of new devices, procedures and drugs [1], being important in research focused on the application and advancement of human and animal welfare [2]. From the 20th century onward, rodents became the main biological model as they provided promising results [3]. Despite such, the translation from animal models to humans has ever since been questioned with regard to its credibility [4] and clinical validity [1], which is attributed to the inherent differences between animal models and human species [1,5].

Despite a high level of genetic homology, as well as several biochemical and physiological similarities, mice diverged from humans more than 85 million years ago. Since then, they have shown physiological and behavioral characteristics that resulted in distinct genotype–phenotype relationships, which probably also led to differences among genotype, microbiome and diseases [4].

In dentistry, several models, such as rodents [6,7], bovine [8], pigs [7,9] and, more recently, fish [10], have been used to evaluate dental caries, periodontal disease and dental materials, including adhesive systems. Despite this, the annual expenditure on laboratory animals, combined with the ethical discussion of the use of animals in research, makes the correct choice of animal model for research become relevant in experimental design.

Animal models allow a closer knowledge on human pathophysiological disease mechanisms, such as drug interactions and immune response evaluation [11]. However, the use of animal models can directly influence the responses of the experiments. In light of these factors, it becomes imperative to conduct a rigorous validation process for the selection of appropriate animal models, taking into consideration the anticipated outcomes and the ethical considerations inherent in their utilization [12].

Actually, one way to infer the best animal model regards the use of bioinformatics tools, as well as evolutionary studies. Phylogeny is the study of the evolution of organisms, genes or proteins. Created in 1866 by the scientist Haeckel with concepts derived from genealogy [13], phylogeny adopted new methods as sequencing data volumes increased. Mutations that occur in a given gene over several generations allow for evolutionary study [14]. These mutations are identified using statistical models of evolution and allow ancestry or descent inference, either through duplication or speciation.

Matrix metalloproteinases (MMPs) are enzymes that are involved in various biological processes of extracellular matrix (ECM) remodeling. Their dysregulation is associated with various pathologies, such as metastasis, arthritis, multiple sclerosis, aneurysm and oral diseases [15,16,17].

MMPs, among other functions, degrade collagen in the dentin matrix [15,16]. Specifically, MMPs act on collagen, which is unprotected by the mineralized matrix, modifying its quaternary and tertiary structure through denaturation and also its primary structure through cleavage [18].

Understanding the action of MMPs in dentin is important for the development of preventive approaches that reduce dentin degradation, providing restorative procedures with greater longevity and bond strength. Despite the increase in research on the action of MMPs in dentin degradation, there is still much to understand and unravel about their functions and biological activities.

There is a continuous effort related to how to organize MMPs and distribute them in classes. Up to the present moment, there are over 20 documented MMPs classes, including collagenases, gelatinases, stromelysins, matrilysins, membrane-type matrix metalloproteinases and unclassified metalloproteinases [19,20].

In dentistry, collagenases (MMPs 1, 8 and 13) and gelatinases (MMPs 2 and 9) are studied in the context of various oral health conditions and procedures. This is because dentin ECM is composed of approximately 90% collagen fibrils. About 98% of dentin collagen is type I and 1–2% is type III, with both preferentially degraded by collagenases, while type V collagen, with about 1%, is preferentially degraded by gelatinases [21]. Collagenases and gelatinases are intrinsic constituents of the fibrillar network of the organic matrix of human dentin and are the most abundant classes of MMPs in this tissue [18,22,23,24].

Curated databases have experienced growth in the last 20 years. The UniProt database, created in 1986, had around 90,000 sequences in 2000 and, in 2022, 568,744 sequences, which accounts for an approximate 630% of data growth [25]. Despite such interest, databases such as UniProt, considered the gold standard in protein functional annotation curation [26], have some problems such as redundancy, inconsistency and uncurated data [27].

The inferences of the function of a gene through bioinformatics tools depend on the correct functional annotation of these genes in databases. Storing incorrect information or records with erroneous annotations can introduce biases into biological analyses [26].

Along with these technical challenges, the high degree of conservation of MMPs represents an additional challenge for their functional annotation and their incorrect identification can invalidate studies.

The aim of this in silico study was to reconstruct the evolutionary history of mammalian MMPs, focusing on collagenases and gelatinases, in order to evaluate the most suitable animal model for research in restorative dentistry. For this, bioinformatics techniques applied to MMP sequences deposited in UniProt were used, evaluating animal models frequently used in restorative dentistry.

## 2. Materials and Methods

**Figure 1:**
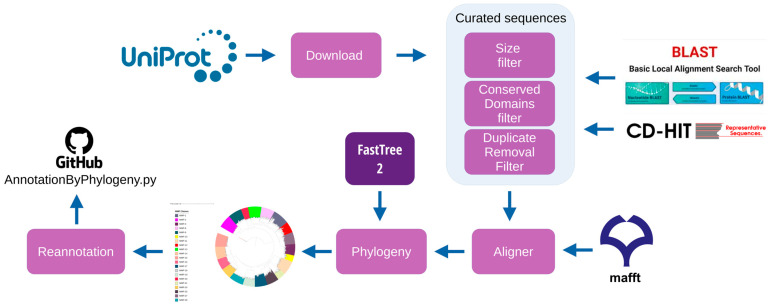
Workflow of this study for identifying the most suitable animal model for use in restorative dentistry.

This study followed a systematic workflow initiated by retrieving mammalian amino acid sequences from MMPs deposited in a public curated protein database (UniProt). Data from multifasta (Multifasta is a common file format used in bioinformatics and molecular biology to store multiple sequences of nucleic acids (DNA or RNA) or amino acids (proteins) in a single file) files of recovered sequences were validated through automatic and semi-automatic processes. The sequences used for phylogenetic reconstruction were selected based on characteristics related to size and the presence of conserved domains. Duplicate sequences were removed. The resulting sequences were then aligned for phylogenetic reconstruction through the maximum likelihood method. MMP sequences that did not correspond to their respective clade/group were re-evaluated and functionally re-annotated (Figure 1).

### 2.1. Retrieved Sequences

Mammalian MMP sequences were retrieved from the UniProt database using the search term based on Kapoor et al. (2016) [28] and are listed below:

- (MMP OR collagenase OR matrix OR metalloproteinase OR metallopeptidase OR (interstitial AND collagenase) OR (neutrophil AND collagenase) OR stromelysin OR metalloelastase OR gelatinase OR matrilysin OR MT-MMP OR (Macrophage AND metalloelastase)) AND (taxonomy_id:40674)

An Archaeal metalloproteinase sequence was chosen as an outgroup in order to aid in adequate MMP phylogenetic tree construction. The outgroup serves as a reference for comparing the ingroup, enabling the rooting of the phylogeny. Since the direction of character change can only be established on a rooted phylogeny, the selection of an appropriate outgroup is crucial for gaining insights into the evolutionary trajectory of traits along the phylogeny [29]. A complete interstitial collagenase sequence was selected and downloaded from the UniProt database as well.

- tr|A0A087RZB5|A0A087RZB5_9ARCH Matrix metalloproteinase-1 interstitial collagenase OS = Marine Group I thaumarchaeote SCGC AAA799-P11 OX = 1502295 GN = AAA799P11_00940 PE = 4 SV = 1
- MLQKKSDEFEQLYEKYDKLKMKVKELSQENQIYSHMCKKIETNSKDLKKSQSNLKKQLDQKLHSQLESEQEKLLLEKQLERSESSSKKSQKKYYVALVMAALSIAIISGAYSIMFAELAGQQYKIEVTPKPTGYTIQNLRGDTINTFLSWRLVPGDTLRVNIINSDNYDPEKIEVIKKTILSEKQLEIDNSLMHKGPKGTTSILYEGWLGALNDASKTDTNLFVPTNIEVIESNNGEGDITIELTNRKNADGFAGWTNSIADDSQNQILKSRITIFAVDSLSLAELETIVRHEMGHALGLAHSTDPEDLMYPTIQTNFPYISECDVDAIESLYDGQNTSEVICEI

### 2.2. Curated Sequences

The mammalian MMP sequences downloaded from UniProt databases were filtered and pre-processed before the construction of the phylogenetic tree multifasta file. Such tasks are described below and comprehend several bioinformatics activities (Table 1).

#### 2.2.1. Sequence Size Filter

We generated a preliminary report with the sequences’ basic statistics (minimum length, maximum length, average length) with an in-house script. After that, sequences with a length within 50% of the average length were kept in order to remove the discrepant sequences that could influence the phylogenetic tree construction.

#### 2.2.2. Conserved Domains Filter

The RPSBlast program [30] was used in order to identify each sequence’s conserved domains. The Conserved Domains Database (CDD) [31] was used as it is a curated and publicly available domains database.

An in-house script was developed in order to search for sequences that have at least three of these specific domains (codes within parentheses are identifiers for the conserved domains in the CDD database):

- matrixin (CDD:395334, CDD:239819, CDD:239806, CDD:239805, CDD:239804, CDD:239803, CDD:239796, CDD:238124 and CDD:214576).
- hemopexin (CDD:395000 and CDD:238046).
- pg-binding (CDD:396175).
- fibronectin (CDD:128373).

#### 2.2.3. Duplicate Removal Filter

The remaining sequences were submitted to the CD-HIT program [32] so that we could remove duplicated sequences (100% identity) and generate a mammalian MMP multifasta file.

### 2.3. MSA Alignment and Phylogenetic Tree Construction

The previously generated mammalian multifasta sequences file was aligned with Mafft (version 7) [33] using standard parameters and later used as an input for the FastTree 2 program (version 2.1.11) [34] for phylogenetic reconstruction, also with default parameters.

### 2.4. MMP Class (Re)annotation

The ITOL software (version 6.8, Biobyte Solutions GmbH, Heidelberg, Germany) [35] was used to identify the MMP classes through different colors. We retrieved each of the available functional MMP classes’ annotations. The MMP sequences that appeared in clades (in phylogenetics, a clade is a group of organisms that includes an ancestor and all of its descendants. Clades are used to represent the evolutionary relationships between organisms based on their common ancestry) with different colors were manually re-annotated.

### 2.5. Conserved Domain Tree

A manual cladogram was constructed to mirror the same relationships between each MMP. A sequence with complete domains was chosen for each MMP and was submitted to Interpro (version 92.0) [36] to locate each domain’s adequate position.

### 2.6. Model Organism Tree

We used only the original sequence’s annotations in order to infer the model organisms’ phylogenetic tree. Specifically, we were interested and restricted our MMP classes’ scope to collagenases (MMP-1, MMP-8 and MMP-13) and gelatinases (MMP-2 and MMP-9) only. The phylogeny was reconstructed using Mafft for multiple alignment [33] and FastTree 2 [34] for the tree reconstruction. For the reconstruction of this phylogenetic tree, only the organisms most used in dentistry were used: *Homo sapiens*, *Bos taurus*, *Mus musculus*, *Rattus norvegicus*, *Canis lupus*, *Oryctolagus cuniculus* and *Sus scrofa*.

## 3. Results

We were able to obtain a total of 176,077 MMP mammalian sequences from the UniProt database. A three-step filtering task was used in order to select sequences that would better suit our study goal. All filters are available and detailed in Section 2.

The first data curation filter was able to remove sequences that could interfere with the multiple alignment. This filter reduced our database to 82,539 sequences. The second filter allowed for identifying sequences with conserved domains characteristic of MMPs, reducing the possibility of identification errors due to poor functional annotation. At the end of this task, this study remained with 3647 sequences. Finally, we applied the third filter, which removed duplicate sequences (100% identity) and ended up with *n* = 3178. Table 2 details all MMP mammalian sequences’ data and statistics.

**Figure 2:**
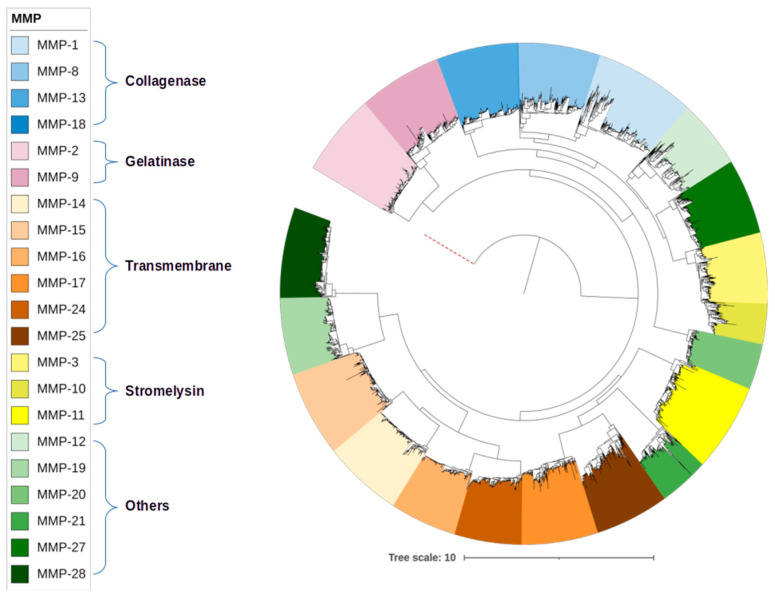
MMP sequence classes’ radial tree (UniProt database). The red dotted line represents the outgroup (Archaea).

We submitted the 3178 MMP mammalian sequences to Mafft for alignment and later reconstructed a radial phylogeny tree with FastTree 2 with a 2000 bootstrap (bootstrap is a resampling technique used to assess the robustness or reliability of the branches in a phylogenetic tree. It is a statistical method that helps estimate how confident we can be in the relationships among taxa (species or other groups) depicted in a phylogenetic tree) with an Archaea metalloproteinase sequence as an outgroup. The MMP classes are identified by a color gradient. We recovered 21 MMPs from collagenase, gelatinase, transmembrane, stromelysins and other classes of MMPs. Among the sequences deposited at UniProt, sequences from MMP-7 and MMP-26 (matrilysins), MMP-23 (transmembrane) and MMP-22 (other MMPs) were not identified (Figure 2).

**Figure 3:**
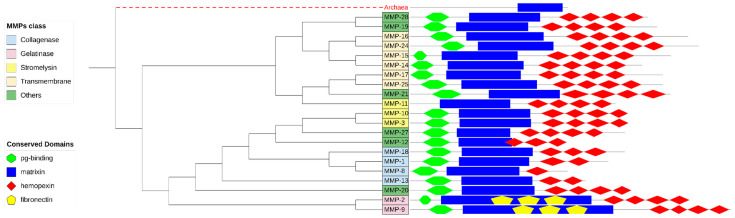
Cladogram of representative sequences and their main conserved domains. The colors represent the classes of MMPs, according to the figure caption.

Figure 3 presents the cladogram constructed from the phylogenetic relationships of the radial tree. The classes of MMPs were represented by a sequence with domains characteristic of each class.

We reannotated several MMP sequences during our study. Specifically, Table 3 shows the number of sequences in each MMP class after the sequence curation process. The table displays both the number of sequences whose annotations were modified due to not being initially annotated as a particular MMP class (Class Annotation), such as Zinc-dependent protein, and the number of sequences whose annotations were altered due to incorrect initial annotations (Reannotation).

**Figure 4:**
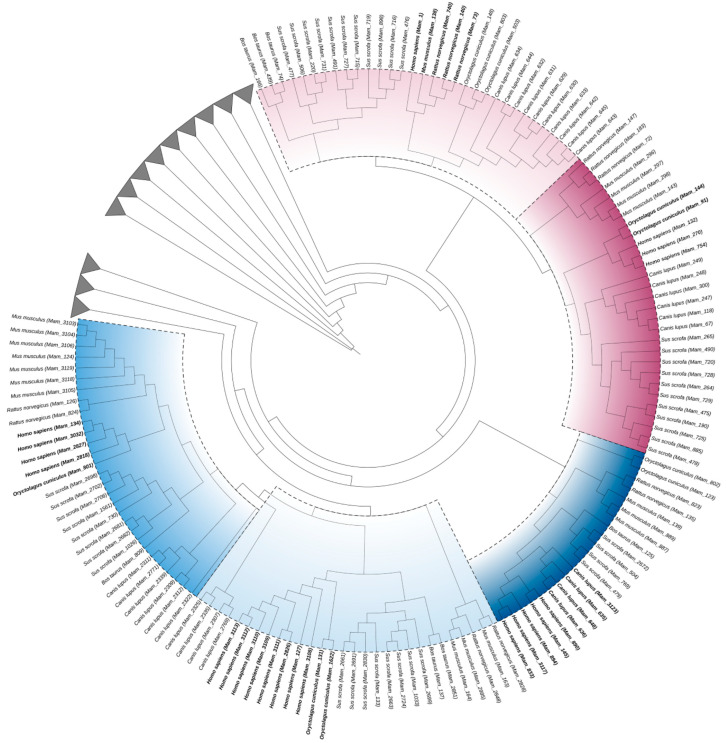
MMP (collagenases and gelatinases) radial tree with main model organisms used in dental research.

The results of our final analysis on how the actual restorative dentistry animal models (*Bos taurus*, *Mus musculus*, *Rattus norvegicus*, *Canis lupus*, *Oryctolagus cuniculus* and *Sus scrofa*), as well as other mammalian organisms, are evolutionarily related to *Homo sapiens* is depicted in Figure 4. As stated, we were specifically interested in collagenases and gelatinases. Thus, the other classes of MMPs were also used in the construction of the radial tree, but these classes were collapsed (clades in gray). The closest organisms to *Homo sapiens* were highlighted at each MMP class.

Our collagenase inference results show that while *Orcytolagus cuniculus* is inferred as the closest organism to *Homo sapiens* in MMP-1, the second closest are *Bos taurus* and *Sus scrofa*.

MMP-8 shows a similar scenario, with *Orcytolagus cuniculus* as the closest organism to *Homo sapiens* and the second closest as *Mus musculus* and *Rattus norvegicus*, both actual dentistry animal models.

MMP-13 depicts *Canis lupus* as the closest to *Homo sapiens* and *Bos taurus* and *Sus scrofa* as the second group of closest organisms.

When it comes to the gelatinases, MMP-2 has *Rattus norvegicus* as the closest to *Homo sapiens*, followed by *Orcytolagus cuniculus*, while MMP-9 has *Orcytolagus cuniculus* as the closest one, second to *Rattus norvegicus* and *Mus musculus*.

## 4. Discussion

Animal models have played a key role in understanding health and disease mechanisms, being indispensable for the development of techniques, drugs and therapeutic approaches. In dentistry, animal models have played a key role in deepening the understanding of syndromes [37] and diseases such as dental caries [38,39] and periodontal disease [40]. Likewise, animal models are used in studies that evaluate the performance of adhesive restorative materials and procedures on mineralized dental tissues (enamel and dentin).

Unlike enamel, the structural and compositional characteristics of dentin make this substrate a challenge yet to be overcome with regard to the longevity of adhesive restorations. Activation of proteolytic enzymes present in the dentine substrate, such as MMPs, can occur under acidic conditions. This can occur in conditions caused by the action of metabolic products of cariogenic bacteria or by treatment of the dentin surface (acid etching) to be restored. Such conditions lead to dentin collagen degradation and consequently compromise the quality of restorative procedures, reducing the longevity of restorations.

In the present study, mammalian amino acid sequences were used to investigate the evolutionary history of matrix metalloproteinases shared between humans and six other organisms: *Bos taurus*, *Mus musculus*, *Rattus norvegicus*, *Canis lupus*, *Oryctolagus cuniculus* and *Sus scrofa*. These species are generally used in dentistry, with the first three being the most used in restorative dentistry [6,7,8]. Although molecular studies can reach down to the subspecies level, this study focused on the species-level results.

MMPs are matrixins that belong to the metzincin family, proteases that have a conserved Met residue in the active site and use a zinc ion in its enzymatic reaction. Members of the MMP family share a core structure that usually consists of a propeptide with approximately 80 amino acids; a catalytic domain of approximately 170 amino acids; a linker peptide of variable length; and a repeat-hemopexin domain of approximately 200 amino acids [41,42,43].

The domain organization and preferred substrate of MMPs are used to classify them into collagenases, gelatinases, stromelysins, matrilysins, membrane-type MMPs (MT) and other MMPs. MMP classes are highly homologous to MMP-1 (collagenase), with the exception of MMP-7 (matrilysin 1) and MMP-26 (matrilysin 2), which lack the linker peptide and hemopexin domain, and of MMP-23, which has a single, cysteine-rich domain and an immunoglobulin-like domain. Despite its elevated homology degree, MMP classes, such as gelatinases (MMP-2 and MMP-9), can present variations, with a catalytic domain that has three type II fibronectin motif repeats [41,42,43].

The importance of MMPs in protein degradation can be evidenced by their wide distribution in different groups of vertebrates [44], including mammals [6,7,8,9], birds [45], reptiles [46,47], amphibians [48] and fish [10], as well as insects [49] and echinoderms [50].

The phylogenetic study of protein families based on amino acid sequences is able to identify their evolutionary history, providing information about their structures, mechanisms and functions. When these proteins share a common descent, the level of sequence similarity is proportional to the similarities in their structures and functions [51].

Genomic data deposited in public databases, even curated, may present integrity, duplication and redundancy problems [52], in addition to errors in their functional annotation [53]. Curating these data before analysis was necessary to ensure the quality of the sequences to be used in the phylogenetic reconstruction.

In the initial statistics, a notably high standard deviation can be observed concerning the sequence lengths (sd = 653) (Table 2). Sequences with highly disparate sizes pose challenges to alignment, resulting in numerous gaps and consequently affecting the phylogenetic reconstruction. The initial filter applied referred to the sizes of the recovered sequences. After calculating the average length of all sequences, only those sequences that were within the specified range of mean +/− 50% were included in this study.

The second filter used was related to the conserved domains of MMPs. MMPs are structures that present high similarity between the different classes, having very characteristic conserved domains, such as (i) the pro-domain (pg-binding) with about 80aa, responsible for keeping MMPs inactive; (ii) the catalytic domain (matrixin) with approximately 160aa; (iii) hemopexin repeat domain; and (iv) fibronectin, exclusive to gelatinases [17,28].

Domains were identified for each sequence, and sequences that exhibited a minimum of three characteristic MMP domains were further considered in this study, which may have excluded the matrilysins.

Finally, the sequences were analyzed for similarity, and those that showed 100% identity with any other sequence in this study were removed.

In this study, 176,077 MMP sequences (Table 2) of different classes were initially downloaded. Over the automated and manual validation steps of the downloaded sequences, this number decreased. In the end, 3178 (1.80%) sequences could be used in the phylogenetic reconstruction.

For this phylogenetic study, widely used and already consolidated tools in Computational Biology were chosen. For multiple sequence alignment (MSA), Mafft was used, which was recently considered one of the best performing tools compared to six other tools of the same function [54]. The method chosen was maximum likelihood (ML) because it is one of the most accurate in terms of phylogenetic inference [55]. The phylogenetic reconstruction tool used was Fasttree 2 for its proven performance in processing files with a large number of sequences [56].

The tree in Figure 2 of this study presents a topology corroborated by the specialized literature regarding the formation of classes of MMPs [28,57,58]. MMPs can be classified according to their structural design or the specificity of their substrates. Kapoor et al. (2016) pointed out that the classification based on the structure of MMPs has been the most accepted and more recent studies point to this same conclusion [59].

Figure 3 shows that the phylogenetic reconstruction obtained is, for the most part, in accordance with the classification by substrate. However, there are some differences such as in stromelysins (MMPs 3, 10 and 11), where, despite the structural design similarity, MMP-11 was positioned in a different clade from MMP-3 and MMP-10, corroborating the studies by [59] which, due to its structure, place MMP-11 close to MMP-28.

Molière et al. (2023) classify MMP-20 as “Other MMPs” due to its structural characteristics. However, phylogenetically in our study, MMP-20 appears close to collagenases (MMPs 1, 8, 13 and 18), according to [60], who described the action of MMP-20 on type V collagen in the dentin matrix.

A similar case occurs with MMP-21, which is classified as “Other MMPs” [28,59], but it is evolutionarily close to transmembrane MMPs 17 and 25, corroborating what was described by [61]. On the other hand, the same authors pointed out that MMP-13 was closer to gelatinases (MMPs 2 and 9) than to collagenases (MMPs 1, 8 and 18), probably due to the low representation of the sequences used by them.

Table 3 scores the sequences that were annotated due to insufficient annotation to correctly classify them into their classes and the sequences that were mistakenly annotated as another class. Phylogenetic methods have been used to functionally re-annotate genes due to the method precision [62,63]. Although the amount of re-annotated sequences may be considered small in relation to the total amount of sequences—7.4% and 0.8%—re-annotation is an important step in order to avoid error propagation [40].

Once the phylogeny confirmed the available literature on MMPs, we began to identify which of the model organisms were closer in evolution to *Homo sapiens* and, therefore, which would be more suitable as an animal model for dental studies. We were able to identify that not all of the chosen model organisms have collagenase and gelatinase MMP sequences deposited at the base of UniProt. Specifically, no MMP-9 sequence was retrieved with regard to *Bos taurus*.

In the field of restorative dentistry, we were able to infer that both *Mus musculus* and *Rattus norvegicus* fit as the closest models for gelatinases (MMP-2 and MMP-9). *Bos taurus* was inferred as the ideal animal model regarding MMP-13 and MMP-1 collagenase classes, while *Mus musculus* and *Rattus norvegicus* stand for ideal results for MMP-8.

Although this study focused on some animal models used in restorative dentistry, the results pointed to other, less used models, such as *Canis lupus* and *Sus scrofa*.

The use of animal models in research has been widely debated due to ethical issues. In addition, there is a great economic impact on maintaining an animal research facility to meet the demand of researchers [64]. On the other hand, the use of animals allows advances in the discovery of new drugs and treatments, placing science as the central point of discussion on this topic [1].

The adequate experimental design, combined with the correct choice of the animal model to be used, is a fundamental issue to balance these two points of view. In this study, we search to evaluate, for each type of collagenase and gelatinase, the model organism that is evolutionarily closest to humans, in order to assist researchers in decision making. However, we have to keep in mind that in vivo tests can result in failures due to uncertainties arising from disparities between species [4].

## 5. Conclusions

This study investigated the evolutionary history of MMPs, emphasizing the importance of accurate data curation and the application of phylogenetic methods to understand the relationships among MMPs. The findings contribute valuable insights to the selection of appropriate animal models for dental research, shedding light on the suitability of specific species for studying different classes of MMPs.

Although we have promising results in choosing an animal model to study MMPs in restorative dentistry, it is important to emphasize that this was an in silico study that evaluated sequences deposited in a public database through phylogeny. In vitro or in vivo future research may demonstrate the effectiveness of the results presented here.

## Figures and Tables

**Table 1:** Downloaded sequences filtering tasks—summary.

Filter	Description
Sequence size	Sequences with a length within 50% of the average length.
Conserved domains	Sequences with at least 3 domains characteristic of MMPs.
Duplicate removal	Removal of sequences with 100% identity.

**Table 2:** Downloaded sequence statistics that were used in the 3-step data curation process.

Statistics/Tasks	Amount Sequences	Smaller Sequence Length	Bigger Sequence Length	Mean	Standard Deviation
Downloaded sequences	176,077	8	14,507	655	653
Sequences above sequence size threshold	82,539	328	984	575	179
Sequences after conserved domains filter	3647	330	980	541	88
Sequences after duplicated sequences	3178	330	980	540	90

**Table 3:** Number of sequences from each class of MMP with the number of sequences that were re-annotated or annotated with specificity.

Class Name	MMP	Amount Sequences	Class Annotation	Reannotation
Collagenases	MMP-1	218	44 (20.2%)	3 (1.4%)
	MMP-8	178	19 (10.7%)	2 (1.1%)
	MMP-13	176	1 (0.6%)	-
	MMP-18	1	-	-
Gelatinases	MMP-2	174	-	-
	MMP-9	180	2 (1.1%)	2 (1.1%)
Transmembrane	MMP-14	171	2 (1.2%)	-
	MMP-15	182	22 (12.1%)	2 (1.1%)
	MMP-16	145	6 (4.1%)	-
	MMP-17	165	17 (10.3%)	-
	MMP-24	144	14 (9.7%)	1 (0.7%)
	MMP-25	160	11 (6.9%)	-
Stromelysins	MMP-3	150	14 (9.3%)	2 (1.3%)
	MMP-10	85	7 (8.2%)	10 (11.8%)
	MMP-11	187	13 (7.0%)	-
Other	MMP-12	145	14 (9.7%)	1 (0.7%)
	MMP-19	162	11 (6.8%)	1 (0.6%)
	MMP-20	96	5 (5.2%)	-
	MMP-21	108	6 (5.6%)	-
	MMP-27	160	8 (5.0%)	3 (1.9%)
	MMP-28	191	19 (9.9%)	-
Total		3178	235 (7.4%)	27 (0.8%)

## Data Availability

The files used in this work are available at https://data.mendeley.com/datasets/8rgh56jjyy/1 (accessed on 30 June 2023).
